# Supplementary material for: The Transcriptome and Metabolome Reveal the Potential Mechanism of Lodging Resistance in Intergeneric Hybrids between Brassica napus and Capsella bursa-pastoris
Source: Int J Mol Sci. 2022 Apr 19;23(9):4481. doi: 10.3390/ijms23094481 (PMC9099622; doi:10.3390/ijms23094481)
Supplement: Supplementary file 1 [file ijms-23-04481-s001.zip › Table S1.pdf]

Table S1: Analysis of G-type and S-type monomers of lignins in the stems of ZY821 and YG689

Stage	Genotype	Acetyl bromide lignin (g g^{-1}dry weight)		G (umol g-1 dry weight)		S (umol g-1 dry weight)		S/G Ratio		G+S (umol g-1 dry weight)	
Seedling	ZY821	0.108 ± 0.004	t-test: 0.1434254	35.2 ± 2.4	t-test: 0.002078301	0	t-test: NaN	0	t-test: NaN	35.2 ± 2.4	t-test: 0.002078301
	YG689	0.117 ± 0.007		96.3 ±7.1		0		0		96.3 ± 7.1	
Bolting/budding	ZY821	0.088 ± 0.001	t-test: 0.03215102	117.9 ± 4.3	t-test: 0.1164725	67.7 ± 2.0	t-test: 0.2693032	0.57 ± 0.05	t-test: 0.01283706	185.6 ± 6.3	t-test: 0.3539799
	YG689	0.106 ± 0.006		151.1 ± 22.1		57.7 ± 11.5		0.38 ± 0.02		208.8 ± 33.6	
Early flowering	ZY821	0.093 ± 0.006	t-test: 0.01037874	89.9 ± 28.1	t-test: 0.01730681	72.8 ± 24.5	t-test: 0.7535377	0.81 ± 0.17	t-test: 0.05400645	162.7 ± 52.6	t-test: 0.08074154
	YG689	0.114 ± 0.004		173.1 ± 19.1		78.5 ± 15.5		0.45 ± 0.06		251.6 ± 34.6	
Terminal flowering	ZY821	0.125 ± 0.005	t-test: 0.01240247	223.4 ± 2.7	t-test: 0.0001762208	169.7 ± 2.7	t-test: 0.002809583	0.76 ± 0.10	t-test: 0.3265369	393.1 ± 5.4	t-test: 0.0005259266
	YG689	0.148 ± 0.007		289.6 ± 4.8		196.8 ± 4.8		0.68 ± 0.07		486.4 ± 9.6	
Mature	ZY821	0.143 ± 0.005	t-test: 0.00713441	1837.5 ± 85.1	t-test: 0.02003187	1178.8 ± 77.4	t-test: 0.002048353	0.64 ± 0.09	t-test: 0.2778509	3016.3 ± 162.5	t-test: 0.001312853
	YG689	0.167 ± 0.002		2306.3 ± 26.7		1729.9 ± 99.4		0.75 ± 0.12		4036.2 ± 126.1	
