# Supplementary material for: The Transcriptome and Metabolome Reveal the Potential Mechanism of Lodging Resistance in Intergeneric Hybrids between Brassica napus and Capsella bursa-pastoris
Source: Int J Mol Sci. 2022 Apr 19;23(9):4481. doi: 10.3390/ijms23094481 (PMC9099622; doi:10.3390/ijms23094481)
Supplement: Supplementary file 1 [file ijms-23-04481-s001.zip › Table S2.pdf]

Table S2. Summary statistics of new gene annotation

Annotated databases	New Gene Number	New Gene annotation Ratio
COG	339	12.57%
GO	1,509	55.95%
KEGG	418	15.50%
Swiss-Prot	1,348	49.98%
NR	2,284	84.69%
All	2,697	
