# Supplementary material for: The Transcriptome and Metabolome Reveal the Potential Mechanism of Lodging Resistance in Intergeneric Hybrids between Brassica napus and Capsella bursa-pastoris
Source: Int J Mol Sci. 2022 Apr 19;23(9):4481. doi: 10.3390/ijms23094481 (PMC9099622; doi:10.3390/ijms23094481)
Supplement: Supplementary file 1 [file ijms-23-04481-s001.zip › Table S3.pdf]

Table S3. 30 DEGs significantly associated to lignocellulose synthesis

Gene alias of *B.napus*	log_2^{FC}	Gene alias of *A.thaliana*	Definition
BnaA02g07630D	6.50	*AT5G58390.1*	peroxidase
BnaA02g09370D	-2.24	*AT3G02020.1*	aspartate kinase 3 (AK3)
BnaA02g16170D	3.81	*AT1G72810.1*	threonine synthase
BnaA02g18920D	6.30	*AT1G78580.1*	trehalose 6-phosphate synthase 1 (TPS1)
BnaA02g19000D	3.49	*AT1G78660.2*	gamma-glutamyl hydrolase 1 (GGH1)
BnaA02g19200D	2.75	*AT1G79230.1*	thiosulfate/3-mercaptopyruvate sulfurtransferase
BnaA06g02800D	-2.01	*AT1G50460.1*	hexokinase 1(HKL1)
BnaA06g07080D	-1.82	*AT1G80740.1*	chromomethylase 1 (CMT1)
BnaA06g37220D	2.24	*AT4G39000.1*	glycosyl hydrolase 9B17 (GH9B17)
BnaA08g09330D	-1.77	*AT4G19810.1*	chitinase (CHIC)
BnaA08g11320D	1.49	*AT4G34200.1*	D-3-phosphoglycerate dehydrogenase 1(PGDH1)
BnaA08g11860D	-1.37	*AT4G33220.1*	pectinesterase 44 (PME44)
BnaA09g42650D	1.68	*AT2G22420.1*	peroxidase 17 (PRX17)
BnaA09g44060D	-3.40	*AT2G19590.1*	aminocyclopropanecarboxylate oxidase (ACO1)
BnaA10g07180D	-6.22	*AT5G54080.2*	homogentisate 1,2-dioxygenase (HGO)
BnaA10g07720D	-1.55	*AT5G51830.1*	fructokinase(FRK7)
BnaA10g15590D	-3.36	*AT5G19550.1*	aspartate aminotransferase 2(ASP2)
BnaA10g24140D	-4.62	*AT4G34710.2*	arginine decarboxylase (ADC2)
BnaC01g05200D	-1.66	*AT4G33030.1*	sulfoquinovosyldiacykglycerol(SQD1)
BnaC01g05230D	1.70	*AT4G33010.1*	glycine decarboxylase P-protein 1 (GLDP1)
BnaC04g11450D	-4.21	*AT5G14800.1*	pyrroline-5-carboxylate reductase (P5CR)
BnaC04g11560D	-6.56	*AT1G43670.1*	fructose-1,6-bisphosphatase (FBP)
BnaC05g47040D	1.30	*AT3G04790.1*	embryo defective 3119 (EMB3119)
BnaC05g48040D	-2.15	*AT3G03780.3*	methionine synthase 2 (MS2)
BnaC05g48310D	2.62	*AT3G02360.1*	6-phosphogluconate dehydrogenase (PGD2)
BnaC05g48570D	5.51	*AT3G01040.2*	galacturonosyl transferase (GAUT13)
BnaC09g10950D	2.63	*AT1G62940.1*	acyl-CoA synthetase 5(ACOS5)
BnaC09g11540D	2.82	*AT2G20420.1*	succinyl-CoA synthetase beta subunit
BnaC09g12180D	4.19	*AT1G64440.1*	UDP-glucose 4-epimerase (UGE4)
BnaC09g14580D	4.51	*AT1G60810.1*	ATP-citratelyaseA-2 (ACLA-2)
