# Supplementary material for: The Transcriptome and Metabolome Reveal the Potential Mechanism of Lodging Resistance in Intergeneric Hybrids between Brassica napus and Capsella bursa-pastoris
Source: Int J Mol Sci. 2022 Apr 19;23(9):4481. doi: 10.3390/ijms23094481 (PMC9099622; doi:10.3390/ijms23094481)
Supplement: Supplementary file 1 [file ijms-23-04481-s001.zip › Table S4.pdf]

Table S4. Identification of significantly different stem metabolites between ZY821 and YG689 by GC-TOF/MS

Metabolite name	Similarity	RT[a]	Mass	VIP	P-value	FC[b]
**at budding stage**						
phytosphingosine	505	24.878	160	3.009	0.001	-20.17
Allylmalonic acid	524	12.593	147	1.397	0.005	-1.31
Farnesal	272	17.534	152	1.422	0.006	-1.46
L-Threose	640	13.982	205	2.664	0.009	-18.53
D-Galactose	475	18.463	204	1.678	0.009	-2.76
Methyl jasmonate	252	17.423	152	1.782	0.010	-1.65
threo-beta-hyrdoxyaspartate	237	15.498	275	2.204	0.016	-1.92
sucrose	802	25.072	192	2.163	0.032	-20.62
D-Glucosaminic acid	500	19.832	319	1.946	0.034	-3.53
L-Valine	279	11.031	156	1.196	0.041	-1.94
beta-Alanine	387	13.724	254	2.157	0.046	-14.13
3-Methyloxindole	280	14.998	176	1.105	0.055	-1.71
raffinose	469	32.369	204	1.066	0.056	-2.29
quinolinic acid	305	16.944	297	1.102	0.058	-1.72
piceatannol	426	27.512	221	1.617	0.060	2.25
3-Hydroxypyridine	642	10.290	152	1.809	0.063	-1.29
myo-inositol	524	20.436	243	1.755	0.067	-1.95
4-Hydroxybenzyl cyanide	238	14.769	191	1.509	0.079	-1.65
ribulose-5-phosphate	482	20.426	138	1.040	0.082	-2.98
Tagatose	606	18.276	160	1.350	0.086	-0.78
**at initial flowering stage**						
Acetol	729	16.787	217	2.293	0.008	-0.34
D-Lyxose	704	15.979	306	1.757	0.012	-2.68
D-Glycerate	455	12.467	116	1.990	0.014	-1.02
Erythrose	328	13.787	89	1.340	0.018	1.18
4-Acetylbutyric acid	314	12.534	61	2.478	0.022	-2.94
L-Valine	279	11.031	156	1.836	0.023	2.51
Citramalic acid	399	14.206	305	1.793	0.023	-0.53
D-erythronolactone	591	15.620	201	1.258	0.027	-1.00
2-Deoxytetronic acid	444	13.803	205	1.577	0.027	-1.90
4-Hydroxyquinazoline	504	15.042	217	1.895	0.029	-24.03
Maleamate	360	13.914	305	2.026	0.029	20.95
4-hydroxybutyrate	590	11.349	205	1.987	0.035	-0.46
Pyruvate	682	8.832	174	1.536	0.037	-2.12
malonate	629	10.908	147	1.107	0.040	0.80
2'-Deoxycytidine 5'-triphosphate degr prod	212	18.381	161	1.139	0.044	1.21
2-hydroxypyridine	636	8.872	240	2.306	0.046	-4.14
serine	568	12.931	204	1.577	0.055	-0.61
Elaidic acid	334	21.552	122	1.774	0.057	1.64

Galactinol	773	28.113	204	1.739	0.058	-0.60
succinic acid	395	12.266	221	1.082	0.062	1.35
1,4-Cyclohexanedione	248	12.148	183	1.528	0.073	-0.62
5,6-dihydrouracil	283	14.507	156	1.766	0.083	2.48
cyclohexane-1,2-diol	539	11.737	129	1.706	0.088	-0.29
mannitol	489	18.993	73	1.020	0.090	0.95
4-hydroxybenzaldehyde	187	14.700	120	1.607	0.093	-0.48
Lyxonic acid, 1,4-lactone	323	17.131	204	1.200	0.098	-0.78
**at final flowering stage**						
Glutaconic acid	504	13.918	233	1.534	0.023	1.10
nornicotine	248	15.11	186	2.633	0.038	4.83
inosine	525	24.364	509	1.457	0.040	1.39
Glucosaminic acid	500	19.832	319	1.405	0.047	-1.62
Leucrose	528	27.225	204	2.087	0.064	0.82
quinolinic acid	305	16.944	297	1.410	0.091	-0.73
2-Methylglutaric Acid	527	13.45	147	1.444	0.100	-1.03

[a]RT = retention time. [b]FC = $\log_2$ YG689/ZY821, the fold change is calculated using the formula log2(YG689/ZY821). YG689/ZY821, mean value of peak area obtained from YG689 /mean value of peak area obtained from ZY821.
