# Supplementary material for: The Transcriptome and Metabolome Reveal the Potential Mechanism of Lodging Resistance in Intergeneric Hybrids between Brassica napus and Capsella bursa-pastoris
Source: Int J Mol Sci. 2022 Apr 19;23(9):4481. doi: 10.3390/ijms23094481 (PMC9099622; doi:10.3390/ijms23094481)
Supplement: Supplementary file 1 [file ijms-23-04481-s001.zip › Table S5.pdf]

Table S5. Identification of significantly different metabolites between ZY821 and YG689 by UPLC-QTOF/MS

MT	name	m/z	RT[a]	ion	VIP	P-value	FC[b]
**Bolting and budding**							
M102T462_2	4-Aminobutyrate	102.055	461.580	(M-H)-	1.599	0.004	0.81
M104T461_2	4-Aminobutyrate	104.071	460.944	(M+H)+	1.616	0.004	0.43
M116T335	D-Proline/L-Proline	116.071	335.340	(M+H)+	1.351	0.030	1.84
M118T415	L-Threonine	118.050	414.810	(M-H)-	1.187	0.031	0.70
M129T643_2	Mesaconic acid	129.018	642.910	(M-H)-	1.770	0.000	0.29
M135T367_2	L-Threonate	135.029	366.910	(M-H)-	1.593	0.002	0.95
M147T519_2	(S)-2-Hydroxyglutarate	147.029	519.294	(M-H)-	1.507	0.009	-0.86
M161T347	D-Alanyl-D-alanine	161.092	346.829	(M+H)+	1.625	0.004	-0.75
M173T643_1	cis-Aconitate/trans-Aconitate	173.008	642.726	(M-H)-	1.695	0.001	0.30
M190T118	Kynurenic acid	190.049	117.551	(M+H)+	1.686	0.002	-0.67
M219T543	5-L-Glutamyl-L-alanine	219.097	543.116	(M+H)+	1.910	0.001	-1.08
M252T116	Deoxyadenosine	252.109	116.275	(M+H)+	1.704	0.008	-1.25
M259T690	Fructose 1-phosphate	259.021	690.477	(M-H)-	1.207	0.039	0.40
M266T141	Adenosine	266.088	140.710	(M-H)-	1.660	0.002	-0.64
M287T268	Kaempferol	287.055	268.114	(M+H)+	1.914	0.000	-2.22
M322T679	CMP	322.043	678.619	(M-H)-	1.767	0.000	1.94
M323T634	UMP	323.027	634.441	(M-H)-	1.707	0.002	2.28
M324T677	CMP	324.059	677.359	(M+H)+	1.799	0.001	1.91
M325T634	UMP	325.043	634.130	(M+H)+	1.719	0.004	2.16
M326T141	Adenosine	326.109	140.649	(M+CH3COO)-	1.669	0.002	-0.70
M360T346_1	Sucrose	360.150	345.861	(M+NH4)+	1.492	0.005	-0.43
M401T525_1	Trehalose	401.128	525.003	(M+CH3COO)-	1.415	0.012	0.56
M489T642_1	CDP-choline	489.114	642.098	(M+H)+	1.403	0.038	1.35

M590T672	GDP-L-Fucose	590.089	672.446	(M+H)+	1.764	0.000	0.90
M71T476	Pyruvaldehyde	71.014	476.389	(M-H)-	1.660	0.001	1.15
**Early flowering**							
M112T220	Cytosine	112.050	219.966	(M+H)+	1.752	0.000	-1.45
M129T643_2	Mesaconic acid	129.018	642.910	(M-H)-	1.544	0.007	0.73
M130T324	L-Pipecolic acid	130.086	324.235	(M+H)+	1.455	0.004	-0.87
M135T367_2	L-Threonate	135.029	366.910	(M-H)-	1.138	0.036	0.77
M147T519_2	(S)-2-Hydroxyglutarate	147.029	519.294	(M-H)-	1.252	0.025	0.95
M150T265	L-Methionine	150.058	265.221	(M+H)+	1.680	0.000	-1.08
M164T215_2	L-Phenylalanine	164.070	215.177	(M+H)+	1.270	0.024	-0.57
M166T213	L-Phenylalanine	166.086	212.838	(M-H)-	1.308	0.015	-0.61
M173T643_1	cis-Aconitate/trans-Aconitate	173.008	642.726	(M-H)-	1.521	0.008	0.76
M190T118	Kynurenic acid	190.049	117.551	(M+H)+	1.325	0.018	-0.65
M195T467_2	D-gluconate	195.049	466.787	(M-H)-	1.179	0.045	-0.76
M219T543	5-L-Glutamyl-L-alanine	219.097	543.116	(M+H)+	1.462	0.001	-0.76
M244T220	Cytidine	244.093	220.121	(M+H)+	1.744	0.000	-1.49
M252T116	Deoxyadenosine	252.109	116.275	(M+H)+	1.543	0.003	-1.13
M258T494	Glycerophosphocholine	258.110	494.378	(M+H)+	1.622	0.000	0.50
M266T141	Adenosine	266.088	140.710	(M-H)-	1.641	0.004	-1.19
M268T140	Adenosine	268.104	139.660	(M+H)+	1.557	0.004	-0.63
M284T244	Guanosine	284.099	244.466	(M+H)+	1.775	0.000	-1.64
M303T141	Uridine	303.082	140.715	(M+CH3COO)-	1.688	0.000	-1.23
M304T348	Cytidine 2',3'-cyclic phosphate	304.032	348.015	(M-H)-	1.592	0.002	-1.39
M306T347	Cytidine 2',3'-cyclic phosphate	306.049	347.380	(M+H)+	1.576	0.002	-1.53
M308T568	Gutathione	308.091	567.709	(M+H)+	1.657	0.001	-1.38
M326T141	Adenosine	326.109	140.649	(M+CH3COO)-	1.630	0.004	-1.20

M330T230	AMP	330.060	229.742	(M+H-H2O)+	1.490	0.005	-1.01
M346T348	GMP	346.055	347.670	(M+H)+	1.530	0.003	-1.13
M360T589	Trehalose	360.150	589.242	(M+NH4)+	1.459	0.002	-0.93
M401T500	Galactinol	401.128	499.964	(M+CH3COO)-	1.211	0.047	-0.36
M405T627	UDP	405.009	627.395	(M+H)+	1.211	0.040	-0.67
M422T628	UDP	422.036	627.754	(M+NH4)+	1.330	0.018	-0.72
M425T410	N,N'-Diacetylchitobiose	425.176	409.572	(M+H)+	1.380	0.007	-1.10
M487T220	Cytidine	487.178	219.666	(2M+H)+	1.723	0.001	-2.36
M522T623	Raffinose	522.203	622.758	(M+NH4)+	1.125	0.040	-0.41
M579T776	UDP-D-glucuronate	579.027	775.928	(M-H)-	1.587	0.003	-0.95
M590T672	GDP-L-Fucose	590.089	672.446	(M+H)+	1.226	0.049	-0.50
M598T736	UDP-D-galacturonate	598.068	735.569	(M+NH4)+	1.290	0.015	-0.54
M598T776	UDP-D-glucuronate	598.068	775.595	(M+NH4)+	1.627	0.002	-0.91
M71T476	Pyruvaldehyde	71.014	476.389	(M-H)-	1.289	0.021	-0.92
**Terminal flowering**							
M104T461_2	4-Aminobutyrate	104.071	460.944	(M+H)+	1.337	0.050	0.47
M117T512_2	Succinate	117.019	511.722	(M-H)-	1.689	0.006	-0.84
M129T643_2	Mesaconic acid	129.018	642.910	(M-H)-	1.903	0.001	0.67
M130T233_2	L-Leucine	130.086	232.908	(M-H)-	1.405	0.034	-0.89
M130T522_2	L-Pyroglutamic acid	130.050	522.029	(M+H)+	1.571	0.021	0.56
M132T209	L-Isoleucine	132.101	208.941	(M+H)+	1.516	0.041	1.31
M132T231_2	L-Leucine	132.101	230.928	(M+H)+	1.345	0.045	-0.79
M146T421	4-Guanidinobutyric acid	146.092	421.190	(M+H)+	1.700	0.009	0.87
M146T523	L-Glutamate	146.045	523.421	(M-H)-	1.559	0.021	0.64
M147T519_2	(S)-2-Hydroxyglutarate	147.029	519.294	(M-H)-	1.440	0.027	0.50
M161T347	D-Alanyl-D-alanine	161.092	346.829	(M+H)+	1.719	0.008	0.34

M173T430	Shikimate	173.044	430.496	(M-H)-	1.294	0.045	-0.58
M173T643_1	cis-Aconitate/trans-Aconitate	173.008	642.726	(M-H)-	1.870	0.001	0.69
M182T294	L-Tyrosine	182.081	294.080	(M+H)+	1.373	0.042	-0.81
M195T467_2	D-gluconate	195.049	466.787	(M-H)-	1.583	0.011	0.91
M203T212	L-Tryptophan	203.081	212.158	(M-H)-	1.570	0.011	-0.63
M205T210	L-Tryptophan	205.097	210.402	(M+H)+	1.415	0.024	-0.54
M243T666	Beta-D-Fructose 6-phosphate	243.026	666.203	(M+H-H2O)+	1.920	0.001	0.81
M243T713	D-Glucose 6-phosphate	243.026	712.940	(M+H-H2O)+	1.469	0.027	0.56
M258T494	Glycerophosphocholine	258.110	494.378	(M+H)+	1.754	0.005	-0.65
M259T690	Fructose 1-phosphate	259.021	690.477	(M-H)-	1.558	0.011	0.46
M277T661	L-Saccharopine	277.139	660.901	(M+H)+	1.497	0.028	0.39
M422T653	UDP	422.036	652.963	(M+NH4)+	1.755	0.014	0.98
M425T410	N,N'-Diacetylchitobiose	425.176	409.572	(M+H)+	2.290	0.000	-2.64
M474T557	Folinic acid	474.173	556.898	(M+H)+	1.593	0.021	0.64
M503T624	Raffinose	503.160	623.735	(M-H)-	1.947	0.001	-0.70
M522T623	Raffinose	522.203	622.758	(M+NH4)+	1.497	0.030	-0.65
M555T55_2	alpha-Linolenic acid	555.441	54.781	(2M-H)-	1.569	0.013	-0.97

[a]RT = retention time. [b]FC = log2 YG689/ZY821, the fold change is calculated using the formula log2(YG689/ZY821). YG689/ZY821, mean value of peak area obtained from YG689 /mean value of peak area obtained from ZY821.
