# Supplementary material for: The Transcriptome and Metabolome Reveal the Potential Mechanism of Lodging Resistance in Intergeneric Hybrids between Brassica napus and Capsella bursa-pastoris
Source: Int J Mol Sci. 2022 Apr 19;23(9):4481. doi: 10.3390/ijms23094481 (PMC9099622; doi:10.3390/ijms23094481)
Supplement: Supplementary file 1 [file ijms-23-04481-s001.zip › Table S6.pdf]

Table S6. Metabolic pathways identified from different metabolites between ZY821 and YG689

Pathway	Bolting stage	Initial flowering stage	Final flowering stage
**TCA cycle**	cis-Aconitate(0.30)	cis-Aconitate(0.76)	cis-Aconitate(0.69)<br>succinate(-0.84)
**Pentose phosphate pathway**		D-gluconate(-0.76)	D-gluconate(0.91)<br>Beta-D-Fructose 6-phosphate(0.81)<br>D-Glucose 6-phosphate(0.56)
**Fructose and mannose metabolism**	GDP-L-Fucose(0.90)<br>Fructose<br>1-phosphate(0.40)	GDP-L-Fucose(-0.50)<br>Mannitol（0.95）	Beta-D-Fructose 6-phosphate(0.81)<br>Fructose 1-phosphate(0.46)
**Galactose metabolism**		Raffinose(-0.41)	Raffinose (-0.70)
**Ascorbate and aldarate metabolism**	L-Threonate(0.41)	L-Threonate(1.35)	
**Purine metabolism**	Deoxyadenosine(-1.25)<br>Adenosine(-0.64)	Deoxyadenosine(-1.13)<br>Adenosine(-0.63)<br>GMP(-1.13)<br>Cytidine (-1.53)<br>AMP(-1.01)	
**Pyrimidine metabolism**	CMP(1.94)<br>UMP(2.28)	Cytosine(-1.45)<br>Cytidine(-2.36)<br>UDP(-0.72)<br>Uridine(-1.23)	UDP(0.98)
**Phenylalanine,tyrosine and tryptophan biosynthesis**		L-Phenylalanine(-0.61)	Shikimate(-0.58)<br>L-Tryptophan(-0.63)<br>L-Tyrosine(-0.81)
**Starch and sucrose metabolism**	Trehalose(0.56)<br>Sucorse(-0.43)	Trehalose (-0.93)<br>UDP-D-glucuronate(-0.95)<br>UDP-D-galacturonate(-0.54)	Beta-D-Fructose 6-phosphate(0.81)<br>D-Glucose 6-phosphate(0.56)
	Mesaconic acid(0.29)	Mesaconic acid(0.73)	Mesaconic acid(0.67)
	2-Hydroxyglutarate	(S)-2-Hydroxyglutarate(0.95)	(S)-2-Hydroxyglutarate(0.50)
