# Supplementary material for: The Transcriptome and Metabolome Reveal the Potential Mechanism of Lodging Resistance in Intergeneric Hybrids between Brassica napus and Capsella bursa-pastoris
Source: Int J Mol Sci. 2022 Apr 19;23(9):4481. doi: 10.3390/ijms23094481 (PMC9099622; doi:10.3390/ijms23094481)
Supplement: Supplementary file 1 [file ijms-23-04481-s001.zip › Table S7.pdf]

Table S7: Primes of the reference gene and detected genes by qRT-PCR

Gene	Primes
Actin	Forward: 5'- CCCTGGAATTGCTGACCGTA-3'
	Reverse: 5'- TGGAAAGTGCTGAGGGATGC-3'
newGene_216	Forward: 5'- TCTGTCTCTCTGATCGGACCT-3'
	Reverse: 5'- TGCATCCCTATATCGCCCAG-3'
newGene_5467	Forward: 5'- CGCTCCGAGTTCATTAGCAG-3'
	Reverse: 5'- TTCGCAGTTGCCTTTCGATG-3'
newGene_875	Forward: 5'- GTCCAAATGCGTCAGATGCTC-3'
	Reverse: 5'- GGTTAAATCCATCCCCAACAACA-3'
BnaA02g18920	Forward: 5'- CACAGAACACTTCCTTCACGTT-3'
	Reverse: 5'- TTCAAGTGCTCGTATAAACCTCT-3'
BnaA06g37220	Forward: 5'- TCACAACTACTCCGATGCTCT-3'
	Reverse: 5'- TAGTAACCGCCAACTAGGTCA-3'
BnaC09g12180	Forward: 5'- CCGGAATGAGCCCTTACGCTA-3'
	Reverse: 5'- TCCATTCAGGATCACCACGTT-3'
BnaA09g42650	Forward: 5'- CCTCCGACCAAGATTCTACAGC-3'
	Reverse: 5'- CCCGTCACATCCATTAACGAAGC-3'
BnaA08g09330	Forward: 5'- TGGCAAGTAACCCAACATCTCG-3'
	Reverse: 5'- TTCATCTCCGTTGCACTACTCG-3'
BnaC01g05230	Forward: 5'- TAACTGCCACCCTCAGACGA-3'
	Reverse: 5'- AGCACACCACACACGTCACC-3'
BnaA08g11320	Forward: 5'- GCTCCACATGCCTCTTACACC-3'
	Reverse: 5'- GCGTCTTCGTCTATCACACCT-3'
BnaA10g15590	Forward: 5'- GATCCAAGCCCATCTAAGCTC -3'
	Reverse: 5'- TTTGTTGAAATCAGCGAGTCCA -3'
BnaA02g16170	Forward: 5'- TCTGGCGTAACCTTTTCGACT -3'
	Reverse: 5'- AAGGTACTGTTTCCCGAACCTC -3'
BnaA08g11860	Forward: 5'- AGCTCCTGAGTACAGCTCGAA -3'
	Reverse: 5'- AGTGGTCCAACCGTCAACGAA -3'
BnaC09g08240	Forward: 5'- TATAGGGAACCCCAACGTGAC -3'
	Reverse: 5'- AATAACACCGCCACCAAGAGC -3'
BnaC04g41120	Forward: 5'- AAGTTGCGACCCCTAAGCC -3'
	Reverse: 5'- TATTCCCCACTCCATAGACGTT -3'
BnaA02g36250	Forward: 5'- CATGAACCCTAACCGTGACCA -3'
	Reverse: 5'- CCACCATCTTCCCGTAGCAA -3'
BnaA08g19210	Forward: 5'- ACATCTTTGAAACGTCGGCAT -3'
	Reverse: 5'- TCCATCTCGCTTAAATTGCCAT -3'
BnaC08g35540	Forward: 5'- TTCTGTGCCAAGCATAATATCGT -3'
	Reverse: 5'- CAGATTTAGCCAAGCGGTCCA -3'
BnaC05g48040	Forward: 5'- TGCTGCCTCTCTCATCACCT -3'
	Reverse: 5'- TGCTGCCTCTCTCATCACCT -3'
BnaA06g07080	Forward: 5'- AAACGTCATTACAGCCAAGCTC -3'

	Reverse: 5’- AAACGTCATTACAGCCAAGCTC -3’
BnaC01g05200	Forward: 5’- TTTTCCGAGCAGCTATAAGCC -3’
	Reverse: 5’- AACCGTCTCCACCAATGACC -3’
BnaA06g02800	Forward: 5’- CTTTGTTATCTGAGCCTTACGTT -3’
	Reverse: 5’- CTCCCTGCTCTTCTTGTCACC -3’
