# Supplementary figures and images for: The Transcriptome and Metabolome Reveal the Potential Mechanism of Lodging Resistance in Intergeneric Hybrids between Brassica napus and Capsella bursa-pastoris
Source: Int J Mol Sci. 2022 Apr 19;23(9):4481. doi: 10.3390/ijms23094481 (PMC9099622; doi:10.3390/ijms23094481)

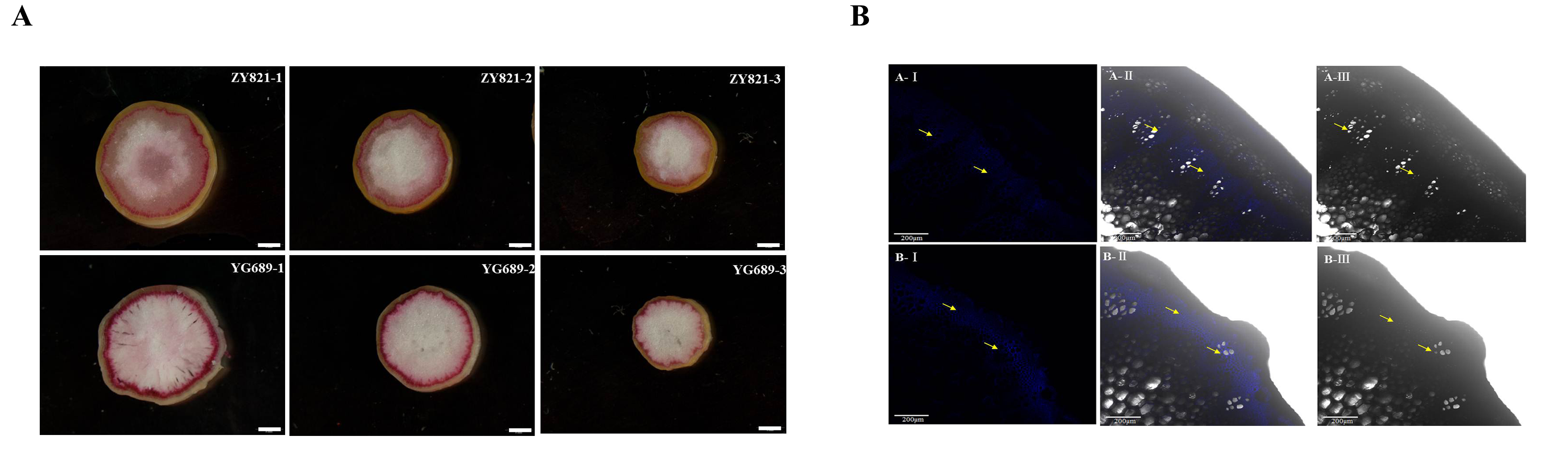

Supplement: Supplementary file 1 [file ijms-23-04481-s001.zip › Figure S1.tif]

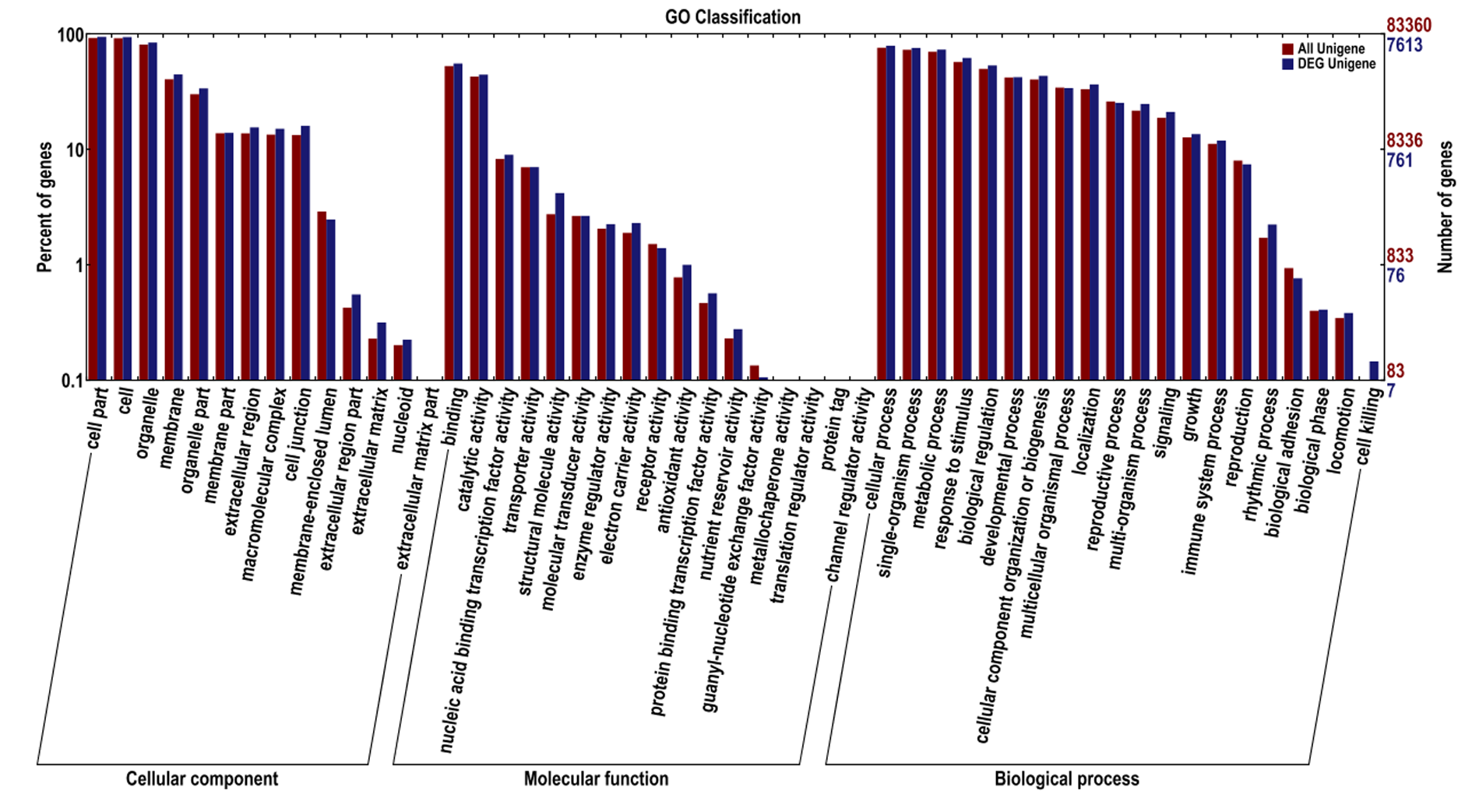

Supplement: Supplementary file 1 [file ijms-23-04481-s001.zip › Figure S2.tif]

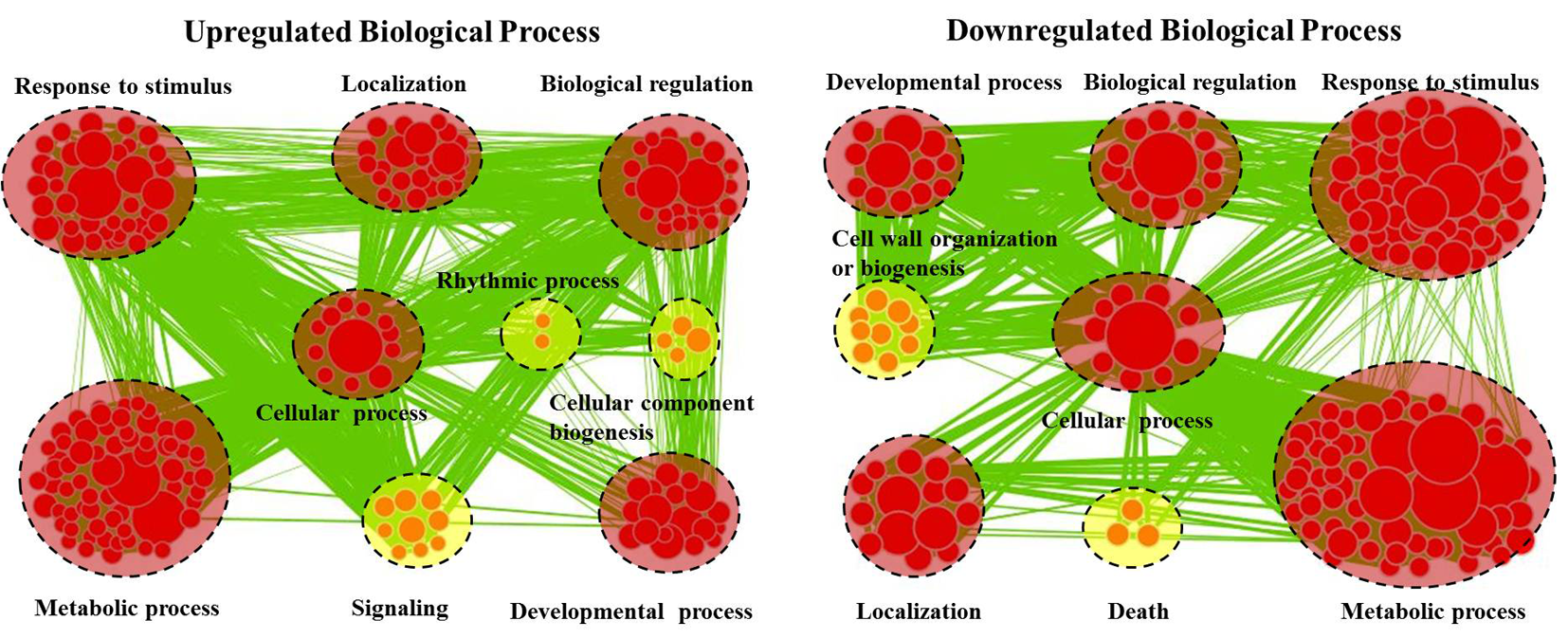

Supplement: Supplementary file 1 [file ijms-23-04481-s001.zip › Figure S3.tif]

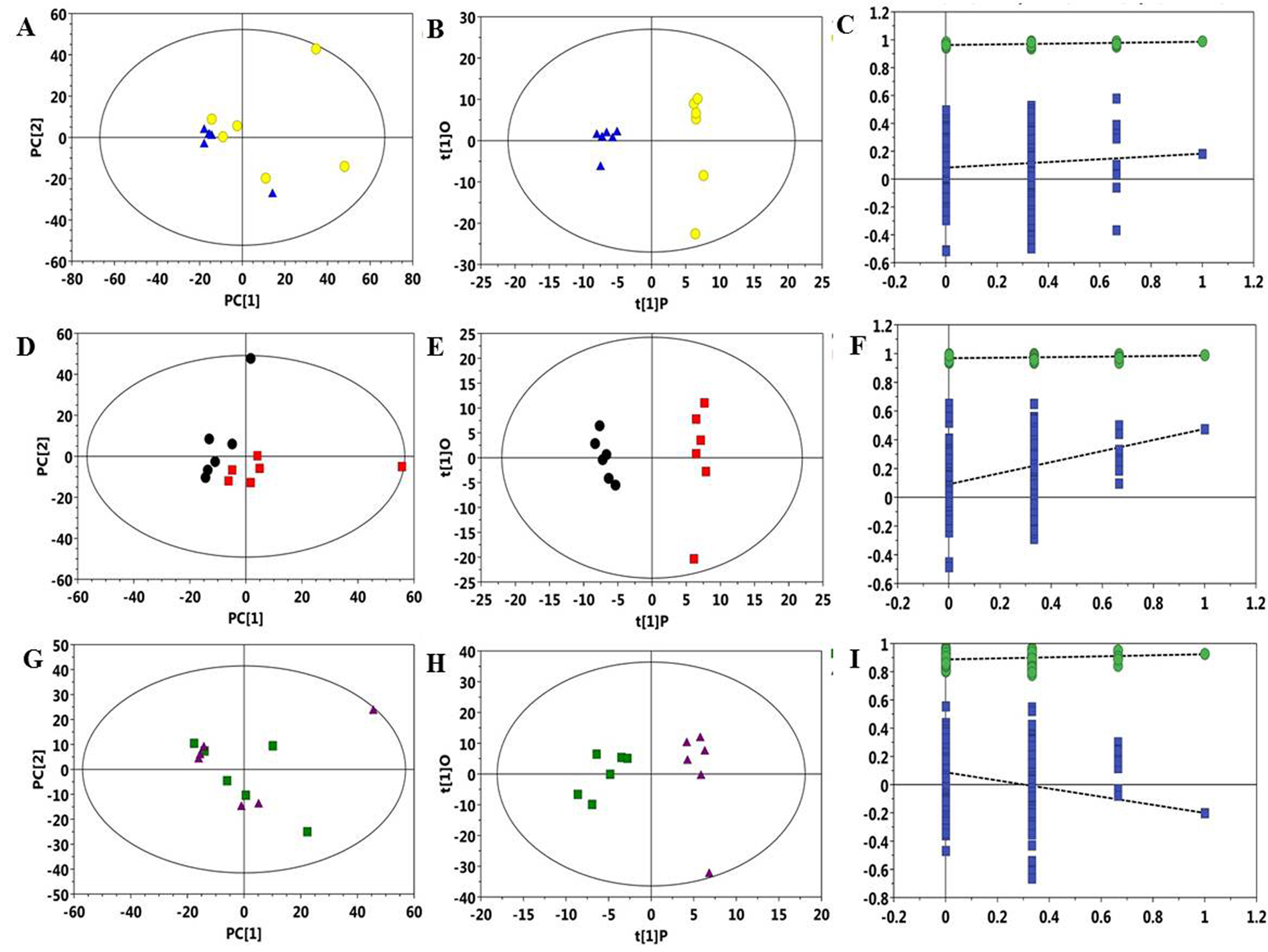

Supplement: Supplementary file 1 [file ijms-23-04481-s001.zip › Figure S4.tif]

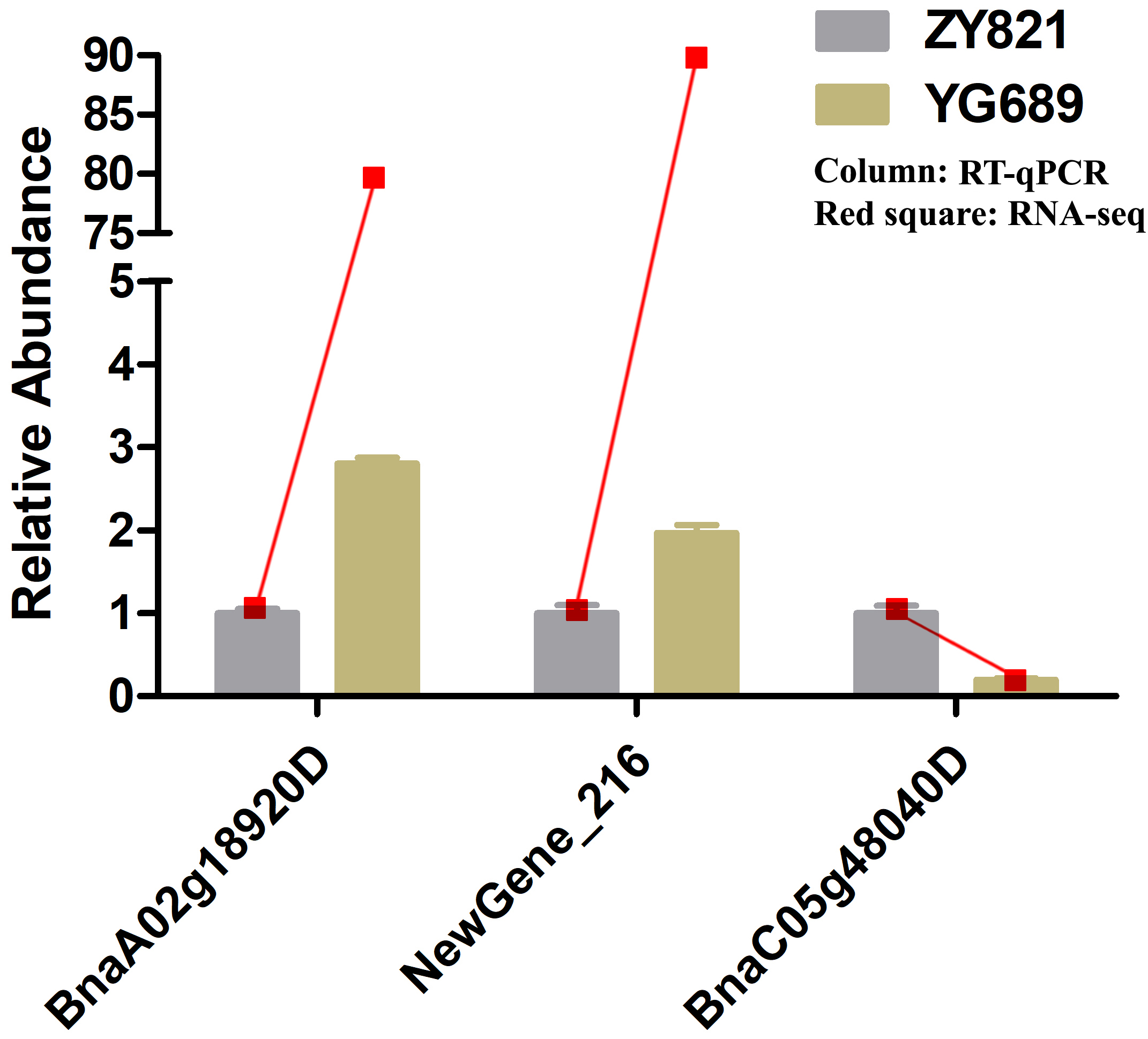

Supplement: Supplementary file 1 [file ijms-23-04481-s001.zip › Figure S5.tif]
